# From intracellular sensors to systemic resilience: Reframing the biology of stress

**DOI:** 10.1016/j.ynstr.2025.100755

**Published:** 2025-09-09

**Authors:** Jakob Hartmann

**Affiliations:** a Department of Psychiatry, Harvard Medical School, McLean Hospital, Belmont, MA, 02478, USA; b Biology of Adversity Project, Broad Institute of MIT and Harvard, Cambridge, MA, 02142, USA

## Abstract

The biological consequences of chronic stress and trauma are complex, influencing multiple systems and contributing to the development of psychiatric disorders such as MDD and PTSD. Yet, the underlying molecular mechanisms that confer susceptibility in some individuals but resilience in others remain incompletely understood. To help close these knowledge gaps, my work centers on glucocorticoid signaling as a core mechanism underlying stress-related adaptations. This includes the glucocorticoid receptor (GR), its co-chaperones FKBP5 and FKBP4, and regulatory partners such as SKA2. Through a combination of genetic, viral, pharmacological, and transcriptomic approaches, my lab has delineated how these molecules influence HPA axis feedback, fear-related learning, and stress recovery. Recently, we identified a novel, GR-independent role for SKA2 in regulating secretory autophagy, a non-lytic autophagy pathway involved in vesicular cargo release, including cytokine secretion in microglia. These findings established a mechanistic link between intracellular stress signaling and neuroinflammatory responses. In a parallel line of research, we are investigating how chronic stress alters the gut microbiome composition and function, and how these changes impact behavior. Our aim is to harness dietary and probiotic interventions to restore homeostatic balance and enhance stress resilience. By integrating molecular neuroscience with immune and microbiome research, my long-term goal is to build a comprehensive, systems-level model of stress vulnerability and resilience. This approach holds promise for identifying novel biomarkers and therapeutic targets that support mental health and resilience across the lifespan.

## 1 Introduction

Severe trauma or chronic stress can profoundly increase the risk of developing psychiatric disorders. A central question in my research, shared by many in the field, is why some individuals maintain psychological resilience while others develop persistent mental health conditions. Disorders such as major depressive disorder (MDD) and posttraumatic stress disorder (PTSD) are not only highly prevalent but also rank among the most disabling globally (Ressler et al., 2022; Cui et al., 2024). Although their impact is well recognized, the molecular pathways underlying these disorders remain incompletely characterized (Fenster et al., 2018; Maddox et al., 2019). Central to the body's stress response are two major systems: the autonomic nervous system, responsible for acute physiological shifts, and the hypothalamic-pituitary-adrenal (HPA) axis, which regulates glucocorticoid release, cortisol in humans and corticosterone in rodents, as a key adaptive mechanism (Ulrich-Lai and Herman, 2009).

While acute glucocorticoid release can be adaptive, facilitating survival and recovery, dysregulated glucocorticoid signaling, particularly under conditions of chronic stress or early-life trauma, can disrupt brain function and increase vulnerability to psychiatric disease (De Kloet et al., 2005; Lupien et al., 2009; Herman, 2022; Kim and Kim, 2023; McEwen and Akil, 2020). Accordingly, impairments in HPA axis regulation represent a shared pathophysiological thread across multiple stress-related conditions.

At the molecular level, glucocorticoid effects are mediated by two nuclear receptors: the mineralocorticoid receptor (MR, encoded by Nr3c2) and the glucocorticoid receptor (GR, encoded by Nr3c1). MRs exhibit high affinity and are predominantly activated under basal glucocorticoid conditions, while GRs become engaged during circadian peaks or in response to stress. This complementary dynamic allows MRs to maintain homeostasis and initiate stress responses, while GRs primarily mediate recovery and feedback inhibition (de Kloet et al., 1998; de Kloet and Joëls, 2024). Disruptions in the MR:GR balance or signaling dynamics of these receptors can alter stress reactivity, fear learning, and emotion regulation which are core features of stress-related psychopathology (Daskalakis et al., 2022).

A central challenge in stress neurobiology is understanding how repeated or traumatic stress becomes biologically embedded, reshaping neural circuits, molecular pathways, and immune tone to promote long-term vulnerability or, in some individuals, resilience.

## 2 From glucocorticoid signaling to neuroimmune crosstalk

From the beginning of my research career, I have been intrigued by the GR and its central role in coordinating stress responses and behavior. My early work, in the lab of Mathias Schmidt at the Max Planck Institute of Psychiatry, Munich, focused on identifying the cell-type and circuit-specific roles of GR in regulating emotion-related behaviors. Although GR is widely expressed in glutamatergic and GABAergic neurons as well as glial cells, its distinct contributions within these populations were not well understood. To further delineate the circuit-specific actions of GR, we generated mice lacking the GR specifically in forebrain glutamatergic or GABAergic neurons (Hartmann et al., 2017). We demonstrated that GR signaling in forebrain glutamatergic neurons, but not GABAergic cell populations, impacts both anxiety behavior and fear extinction behavior. Moreover, we showed that selective GR signaling within the basolateral amygdala excitatory cell population mediates fear learning and fear extinction but, importantly, does not impact innate anxiety behavior. This clarified a long-standing question about the distinct behavioral domains regulated by GR across neuronal subtypes and highlighted glutamatergic GR signaling as a potential therapeutic target for fear-related disorders.

Although the GR is essential for mediating glucocorticoid signaling and HPA axis feedback, it functions as part of a larger protein complex that includes multiple co-chaperones (Binder, 2009; Grad and Picard, 2007). I became increasingly interested in how these co-factors modulate GR activity, as their more subtle regulatory effects may offer promising therapeutic entry points. In parallel with my work on GRs, I began investigating two key co-chaperones: FKBP5 (FK506 binding protein 51) and FKBP4 (FK506 binding protein 52). FKBP5 acts as a negative regulator by decreasing GR sensitivity and nuclear translocation, while FKBP4 supports GR activity by promoting its nuclear import (Zannas et al., 2015; Wochnik et al., 2005). At the time, FKBP5 had been identified as a genetic risk factor for stress-related disorders such as MDD and PTSD (Binder, 2009; Binder et al., 2004, 2008; Klengel et al., 2013; Willour et al., 2009), yet few preclinical studies had validated its role in disease-relevant outcomes (O'Leary et al., 2011; Scharf et al., 2011; Lee et al., 2010; Touma et al., 2011). Our work helped elucidate the distinct roles of FKBP5 and FKBP4 in modulating stress-induced neuroendocrine responses and behavioral outcomes relevant to psychiatric illness. We demonstrated that *Fkbp5* knockout mice exhibited reduced sensitivity to both acute stress and chronic social defeat, particularly in terms of behavioral and neuroendocrine responses (Hartmann et al., 2012a; Hoeijmakers et al., 2014; Gassen et al., 2014). In contrast, mice lacking *Fkbp4* showed the opposite phenotype (Hartmann et al., 2012b). Using both viral and pharmacological tools, we further showed that *Fkbp5* signaling in the amygdala modulates anxiety-like behavior, supporting FKBP5 as a promising target for therapeutic intervention (Hartmann et al., 2015; Gaali et al., 2015).

More recent studies have begun to dissect the cell type-specific, circuit-level, and sex-dependent roles of FKBP5 in stress-related behaviors and GR signaling, providing a more detailed and differentiated understanding of its function in distinct neural populations (Brix et al., 2022; Engelhardt et al., 2021a, 2021b; Kovarova et al., 2024; Sabbagh et al., 2014). For example, *Fkbp5* deletion in Sim1^+^ PVN neurons reduces acute stress reactivity and enhances GR sensitivity, highlighting a key role in HPA axis control at the circuit level (Häusl et al., 2021); conditional knockouts in glutamatergic versus GABAergic neurons produce opposite behavioral effects in a sex-specific manner (van Doeselaar et al., 2023); and FKBP5 in forebrain excitatory neurons mediates the long-term, pro-resilient effects of early life stress in females but not males (van Doeselaar et al., 2025). These findings underscore the nuanced and context-dependent nature of FKBP5 signaling in brain and behavior.

We also established a link between FKBP5 signaling and autophagy pathways, identifying a molecular mechanism through which FKBP5 influences antidepressant treatment response across cellular, animal, and human models (Gassen et al., 2014, 2015). In this context, FKBP5 expression levels may serve as predictive biomarkers for antidepressant efficacy, offering potential clinical utility beyond its genetic associations.

During my postdoctoral training in Kerry Ressler's lab at McLean Hospital, I expanded my research to explore how not only the GR, but also the MR, interact with FKBP5 - focusing in particular on the concept of MR:GR balance in the hippocampus (de Kloet and Joëls, 2024; Daskalakis et al., 2022). We found that MRs regulate baseline FKBP5 expression, which in turn sets the sensitivity of GR signaling under acute stress (Hartmann et al., 2021). This work revealed an MR-FKBP5-GR feedback loop that fines-tunes hippocampal glucocorticoid responsiveness, providing new insight into how MR signaling, often underappreciated in stress research, contributes to psychiatric disease risk.

Upon establishing my independent lab, I focused on understanding how stress signaling is regulated in the brain beyond traditional HPA axis components. We were the first to identify a novel function for SKA2 (spindle and kinetochore-associated complex subunit 2), a gene previously recognized solely for its function in cell cycle regulation (Hanisch et al., 2006), in modulating stress signaling in the brain. Although SKA2 had been identified as a GR interactor in peripheral dividing cells (Rice et al., 2008) and had been associated with stress-related psychiatric disorders including PTSD and suicide risk (Sadeh et al., 2016a, 2016b; Kaminsky et al., 2015; Boks et al., 2016; Pandey et al., 2016), its function in nonreplicating, postmitotic neurons, and its potential role in regulating HPA axis activity, had remained unexplored. Our research demonstrated that SKA2 promotes GR nuclear translocation by facilitating FKBP4 binding and dissociating FKBP5 from the GR-HSP90 complex (Hartmann et al., 2024a). This mechanism is essential for maintaining HPA axis responsiveness and allostasis, key processes implicated in stress-related psychiatric disorders such as bipolar disorder (BD). Notably, we observed altered SKA2 expression in the hippocampus and amygdala of individuals with BD, suggesting that SKA2 may contribute to disease-relevant dysregulation of glucocorticoid signaling. Together, these findings reveal SKA2 as a previously unrecognized modulator of stress responsiveness in the brain and a potential target for therapeutic intervention.

Building on this GR-focused framework, in close collaboration with Nils Gassen and his lab at the University of Bonn, we uncovered a second, GR-independent role for SKA2 in regulating secretory autophagy, a non-lytic vesicle-mediated process by which pro-inflammatory cytokines such as IL-1β are released (Ponpuak et al., 2015; Dupont et al., 2011; Kimura et al., 2017). We found that SKA2 inhibits FKBP5-driven IL-1β secretion through suppression of secretory autophagy (Hartmann et al., 2024b; Bajaj et al., 2024). This regulation appears especially relevant in microglia, where stress alters autophagic and cytokine pathways. These results expand the functional landscape of SKA2 and FKBP5, identifying them as multi-function proteins that coordinate glucocorticoid signaling and inflammatory responses through distinct intracellular mechanisms. In addition to its roles in stress hormone signaling and neuroinflammation, FKBP5 also contributes to systemic metabolic regulation, reinforcing its status as a pleiotropic stress-responsive protein. Studies have shown that FKBP5 modulates Akt signaling, glucose uptake, and insulin sensitivity, and is implicated in adipocyte differentiation and energy metabolism-linking chronic stress to metabolic dysfunction and systemic inflammation (Balsevich et al., 2014, 2017; Häusl et al., 2019, 2022; Sidibeh et al., 2018; Pereira et al., 2014; Zannas et al., 2019; Hähle et al., 2019; Smedlund et al., 2021). This conceptual shift, from stress hormone regulation to immune modulation, has laid the foundation for my lab's current research program, which aims to understand how chronic stress drives neuroimmune dysfunction and psychiatric disease risk.

## 3 Current work

In 2023, I established the Hartmann Lab at McLean Hospital, Harvard Medical School. Our research aims to map how intracellular stress signaling pathways, particularly GR, FKBP5, FKBP4, and SKA2, interact with immune and microbial systems to shape long-term stress outcomes. Our work spans genetically engineered mouse models, transcriptomics, behavioral phenotyping, microbiome profiling and human postmortem tissue analysis. Through this multidisciplinary lens, we aim to build an integrated model of stress biology that links intracellular dynamics to whole-organism and population-level outcomes.

### 3.1 Secretory autophagy: a molecular bridge between stress and neuroinflammation

A growing body of evidence implicates neuroinflammatory processes in the etiology of stress-related psychiatric disorders such as PTSD and MDD. Both epidemiological and preclinical studies indicate that chronic stress alters microglial function, priming the brain's immune system toward hyper-responsivity. Yet the intracellular mechanisms linking neuronal stress signaling to immune activation remain incompletely defined.

Our lab is investigating secretory autophagy as one such mechanism. In contrast to canonical degradative autophagy, secretory autophagy facilitates the extracellular release of cytokines and other vesicular contents. Given that both FKBP5 and SKA2 modulate this pathway, we hypothesize that stress-induced transcriptional changes in these genes alter the brain's inflammatory set point. Using molecular assays, region-specific knockouts, and chronic stress paradigms, we are identifying molecular checkpoints at which secretory autophagy becomes dysregulated. Our goal is to determine how this pathway contributes to behavioral pathology and to uncover novel targets for immunomodulation in stress-related disease.

### 3.2 Expanding toward a holistic view: microbiome-based interventions

In parallel, our lab is investigating a distinct but complementary axis: the role of the gut microbiome in shaping stress responses and resilience. While initially viewed through the lens of metabolic health, the gut microbiome is now understood to regulate immune, endocrine, and neural systems, making it a powerful potential modulator of stress-related behavior. Although its specific role in stress-related psychiatric disorders remains incompletely defined, there is increasing evidence that microbial composition influences HPA axis activity, anxiety-like behavior, and cognition (Ke et al., 2023). We aim to identify how stress-induced shifts in microbial composition affect brain function and behavior, and test whether dietary and probiotic interventions can restore microbial balance and improve molecular and behavioral outcomes. These efforts reflect a broader transition in our lab's approach, from circuit-specific mechanistic studies to systems-level investigations that encompass both central and peripheral contributors to stress vulnerability. In doing so, we aim to build a more holistic model of stress-related disease and resilience.

## 4 Looking ahead: toward a systems-level model of stress

Moving forward, the field of stress neurobiology must transition from a compartmentalized view to one that embraces the full complexity of multisystem interactions. While past work has yielded important insights into neurocircuitry, immune signaling, endocrinology, and peripheral physiology, these domains are increasingly recognized as interdependent. Chronic stress disrupts not only brain function but also the communication between central and peripheral systems, including the immune system, endocrine pathways, and gut microbiota. A new framework is needed, one that captures this dynamic interplay and reflects the real-world complexity of stress-related disorders.

Future progress will depend on our ability to map bidirectional signaling across brain and body. For instance, stress-induced changes in microbial composition can influence brain function via cytokine production, vagal signaling, and microglial activation. Conversely, central neuroendocrine responses can reshape the gut and immune landscape. Identifying biomarkers that reflect these integrated states, and that can be tracked across development and context, may offer a path toward precision psychiatry. Systems biology and high-throughput approaches will be essential, as will computational modeling to uncover causal links and regulatory networks.

To complement this systems-level approach, it will be critical to account for both cell-type-specific and sex-specific effects. Emerging evidence highlights that stress responses are shaped by distinct cellular populations, including specific neuronal subtypes, glial cells, and immune effectors, as well as by biological sex (Cathomas et al., 2022; Bangasser and Wicks, 2017; Heck and Handa, 2019). These factors influence not only susceptibility and resilience but also treatment responses. My lab aims to incorporate cell-type specific manipulations and analyses, and sex-balanced experimental designs to systematically dissect these variables. This work will help build a more granular and inclusive understanding of stress biology, better reflecting the heterogeneity seen in clinical populations.

Translational relevance will also require closer integration between animal models and human data. Transcriptomics, digital phenotyping, longitudinal behavioral tracking, and biospecimen collection in diverse populations can help bridge this gap. Ultimately, advancing our understanding of stress biology will require sustained collaboration across disciplines. Neuroscience alone cannot solve the puzzle of adversity; it must be joined by psychiatry, endocrinology, immunology, microbiology, data science, and other disciplines.

One initiative that exemplifies this approach is the Biology of Adversity Project at the Broad Institute of MIT and Harvard (https://www.broadinstitute.org/biology-of-adversity-project). This collaborative effort brings together researchers from across domains to understand how stress and adversity shape biological systems from molecular signaling and circuit function to population health. By integrating data across species, tissues, and developmental stages, the project aims to identify common biological mechanisms that underlie vulnerability and resilience. As a contributing investigator, I am particularly focused on linking mechanistic insights such as GR dynamics and neuroimmune regulation, to broader frameworks that account for human diversity, environmental exposures, and early-life programming.

Our ongoing and future work seeks to illuminate how intracellular stress sensors such as FKBP5 and SKA2 participate in multisystem adaptation, how microbiota-derived signals influence brain function, and how molecular pathways converge on resilience or risk. By embracing integrative and collaborative models, we hope to move the field toward interventions that are both biologically grounded and clinically impactful, supporting resilience across life stages, diagnoses, and populations.

## 5 Concluding remarks

Stress-related psychiatric disorders arise from a complex web of molecular, cellular, and systemic dysregulation. To fully understand this web and to identify tractable points for intervention, we must integrate insights from stress hormone signaling, immune responses, and environmental influences. My work has traced a path from intracellular GR modulators like FKBP5 and SKA2 to broader questions about how stress is encoded across tissues and timescales. As the field continues to evolve, I believe that embracing cross-disciplinary, systems-level approaches will be key to advancing both basic understanding and clinical translation. By investigating the molecular underpinnings of resilience, we move closer to developing interventions that not only mitigate pathology, but strengthen the capacity to adapt. The next generation of stress research will depend not only on understanding vulnerability, but on uncovering the molecular signatures of resilience and transforming them into tools for prevention and repair.

**Figure:**
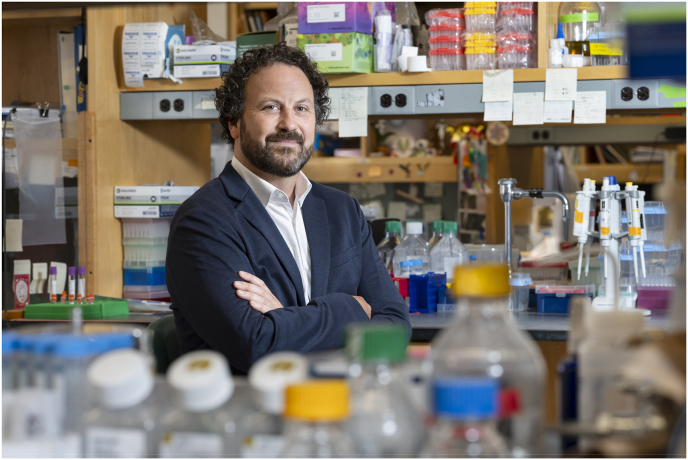

## Declaration of competing interest

The author declares no competing financial interests or personal relationships that could have appeared to influence the work reported in this review.

## Data Availability

No data was used for the research described in the article.
